# Book Reviews

**Published:** 1911-09

**Authors:** 


### BOOK REVIEWS

DISEASES OF THE STOMACH. WITH SPECIAL REFERENCE TO TREATMENT. By Charles D. Aaron, Sc. D., M. D., Professor of Gastroenterology and Adjunct Professor of Dietetics in the Detroit College of Medicine; Professor of Diseases of the Stomach and Intestines in the Detroit Post-Graduate School of Medicine, etc. Octavo, 555 pages, with 42 illustrations and 21 plates. Cloth, 1911.

The author's objective has been to write a book on the purely practical aspects of gastric diseases, to limit himself to what is definitely known and to exclude mere speculation. Etiology, symptomatology, pathology and diagnosis are introduced so far as necessary to an understanding of the methods of treatment proposed, and space is therefore available for developing the therapeutic sections in full detail. Diseases of the stomach are so universal and are the starting point of so many other evils that every physician needs to be posted on the latest advances attained in this highly progressive branch of medicine. These he will find authoritively set forth in Professor Aaron's pages, together with previous knowledge that has stood the test of time, in a word, the whole subject as understood at the present date. He has given the indications for surgical intervention, so that this recourse may be utilized only when necessary, and useless or injurious operations may be avoided. The author's very eminent position in the ranks of American gastro-enterologists will attract wide attention to this important work, which will not disappoint expectations.

---

HOOKWORM DISEASE. Etiology, Pathology, Diagnosis, Prognosis, Prophlylaxis, and Treatment. By George Dock, A. M., M. D., Professor of the Theory and Practice of Medicine, Medical Department Tulane University of Louisiana, New Orleans, and Charles C. Bass, M. D., Instructor of Clinical Microscopy and Clinical Medicine, Medical Department Tulane University of Louisiana, New

Orleans. 250 pages, royal octavo. Fifty illustrations, including one colored plate. Price $2.50. C. V. Mosby Company, St. Louis, Publishers.

This is a strange and important disease and particularly important in the South where its ravages are so great. It has probably existed since remote antiquity among tropical people. Dock has gone thoroughly into the subject and his book should be in the hands of every practitioner.

---

PROGRESSIVE MEDICINE. A Quarterly Digest of Advances, Discoveries and Improvements in the Medical and Surgical Sciences. Edited by Hobart A. Hare, M. D., assisted by L. L. Appleman, M. D. Volume III. Lea & Febiger, Philadelphia.

Diseases of the Thorax and its viscera, including the heart, lungs and blood vessels are discussed by William Ewart, M. D. Dermatology and Syphilis are taken up by William Gottheil, M. D. In his discussion of 606, we are quite surprised that Gottheil favors the intramuscular injection of this remedy, when, as he admits, the pain is often so severe the patient refuses to take further treatment, which often is needed and should always be recommended. Ehrlich says the "intravenous is to be preferred to all other treatment, although the technical difficulties of administering it will be an obstacle in the introduction of the remedy into general practice." He further says, "The interests of the patient demand that only the most efficient form of treatment should be decided upon."

The subject of obstetrics is covered by E. P. Davis, M. D., and diseases of the nervous system by W. G. Spiller, M. D.

---

HOSPITAL MANAGEMENT. Edited by Charlotte A. Aikens. Illustrated. W. B. Saunders Co., Philadelphia.

The purpose of this book is to promote system, economy, and a better understanding of the principles that underlie successful, efficient hospital management. Different chapters are written by the superintendents of the large hospitals of the United States and Canada.

